# Self-Reported Hepatitis B Vaccination Uptake and Associated Factors Among Adults Attending Budwale Health Center in Mbale District Uganda

**DOI:** 10.24248/eahrj.v7i2.739

**Published:** 2023-11-30

**Authors:** Naziru Rashid, John Bosco Ddamulira, Steven Kabwama Ndugwa, Namutundu Juliana, Ssentogo Julius, Alex Daama, Aidah Ajambo, Namatovu Josephine, Felix Mutaryebwa, Ronald Ahumuza, James Batte, Faridah Nakayiza, Mariam Abbasi Ndagire

**Affiliations:** a Department of public health, Islamic University in Uganda; b Department of Epidemiology and Biostatistics, Makerere University School of Public Health; c Uganda. National Health Laboratory Services (UNHLS), Ministry of Health Uganda; d Rakai Health sciences program; e Mayuge Institute for Global Health Sciences Research and Innovation Limited; f Department of Biomedical Sciences, Kampala International University; g Habiib Medical School, Islamic University in Uganda; h Department of Disease Control & Environmental Health, School of Public Health, College of Health Sciences, Makerere University, Kampala, Uganda

## Abstract

**Introduction:**

The introduction of Hepatitis B vaccination in the general community requires understanding the factors that determines its uptake. This is specifically essential for planning, implementation and evaluation of the effectiveness of the program. This study investigated the uptake and associated factors of Hepatitis B Vaccination among clients attending Budwale Health Center in Eastern Uganda.

**Methods:**

Facility based cross-sectional study was conducted from July to December, 2022. Interviewer administered questionnaire was used to collect data from 403 respondents who were aged 18 years and above attending a government health facility. Data was analyzed using SPSS version 20. Vaccination uptake was determined by descriptive statistics, measures of association were done using modified Poisson regression to estimate the association between the factors and vaccination uptake. Scores on knowledge questions were categorized using bloom's cut off point into good knowledge (>80%), moderate knowledge (60–79%) and poor knowledge (< 60 %).

**Results:**

Vaccination uptake was at 68.7% which is the recommended 90% required to achieve herd immunity. Slightly above half of the participants (51.8%) and 35.7% had moderate and good and knowledge about HBV vaccination and infection respectively. Age category (28–37 years) were more likely to be vaccinated than other age categories. PR=1.21 95% CI: (1.025, 1.418) *P=0. 024*. Clients who had moderate knowledge were more likely to be vaccinated compared to those with poor knowledge PR=2.81, 95%CI: (1.830, 4.306) *P=0.001*. The study also found that the cost of vaccines, presence of health workers, distance of health facilities from the home, long waiting queues and occupational risks were the main reasons for low uptake of vaccine.

**Conclusion and recommendation:**

The uptake of HB vaccination was moderate among the studied group which was influenced by individual factors such as knowledge on HB as well as health services access factors. Therefore, due attention should be given to filling the knowledge gaps through education and sensitization, and addressing the barriers to accessing vaccination services.

## BACKGROUND

Hepatitis B is a viral infection caused by Hepatitis B virus (HBV) that attacks the liver and can cause both acute and chronic disease. Globally it is estimated that 296M people are living with Chronic form of the infection while 1.5million new cases are identified annually.^1^

Africa is one of the disproportionately affected continents with approximately 81million people chronically infected with HBV.^2^ Uganda with a 4.3% national prevalence of hepatitis B is one of the high burdened countries.^3^ This prevalence varies across the different regions of the country and Mid-Eastern Uganda where Mbale belongs has an HBV prevalence of 2.1 %, and the Bagisu and Sabin who are the main tribes with in Mbale are estimated to have a prevalence of 3.1%.^4^ According to the MoH 2018 report from Uganda Blood Transfusion Services, (UBTS) Mbale had an HBV prevalence of 4.21% among the blood donors.^4^

HBV is usually transmitted through getting in contact with infected body fluids such as blood, sexual fluids and its spread can be mitigated via simple approved interventions such as injection safety and vaccination against the virus. The vaccines are safe, available, and effective hence providing long life immunity to the negative individuals that complete the dosage as required.^5^ This inactivated vaccine works by stimulating the innate immune system to produce antibodies against HBV hence providing immunity to the body against HBV.^6^ The HB vaccine is also known as the first anti-cancer vaccine since it protects against acquisition of HB which leads to development of hepatocellular carcinoma as its late complication.

In the efforts of ending HB as a global threat; the WHO embarked on a campaign to eliminate Hepatitis by 2030. This is postulated to be achieved via different avenues which include risk reduction counselling and disease awareness, prevention of mother to child transmission of HB strategies and vaccination among others.^7^

Vaccination is the process of introducing a substance (especially a vaccone) into a person's body with the goal of preventing them from getting a particular disease. Several studies have indicated varying levels of vaccination uptake for example a study done among healthcare workers at a national hospital in Tanzania found uptake at 33.6%.^8^ Another study among the medical students at Makerere university found uptake at 44.3%.^9^ A study done among the dental surgeons in Benin city Nigeria indicated that only 20 % of the respondents had received 3 doses of HB vaccination.^10^ Uptake among adult patients in south west Poland was found to be 36.4%.^11^ While that among commercial sexual workers in Netherlands was at 63%.^12^

Furthermore, a study done among the health workers at the University of Nigeria Teaching hospital showed an uptake of HBV Vaccination at only 14.2%.^13^ Similar studies in Juba teaching hospital and among healthcare providers in Wakiso district in Uganda found HB vaccination uptake at 44.2% and 57.8% respectively.^14,15^ Regarding knowledge about HB vaccination and infection, a study done among the health care workers in Lagos state hospital showed good knowledge on HB vaccination.^16^ A similar study done by Rashid and Swaibu found moderate knowledge on HBV infection and HB vaccination however this study did not tell us about the uptake of HB vaccination.^17^

Among the factors for vaccination uptake included cost of vaccines, long vaccination schedules, and lack of time, occupational exposure and knowledge were among the factors associated with HB vaccination.^10,13,18^ Most of the available literature on uptake of HB vaccination and the associated factors is on health care providers and medical students. There was scarcity of information about the general population. However, for the HB vaccination to be effective in controlling the spread of diseases, a significant number of people in the community including both health workers and non-heath workers must be vaccinated to achieve herd immunity.^19^ Despite the interventions by the MoH in the recent years, vaccination uptake in Mbale District continue to be undocumented. Unavailability of information on vaccine uptake hinders evidence-based planning of implementation which could lead to increase in cases of HBV infections and ultimately to associated complications of liver cancer and cirrhosis.

The current study therefore determined the prevalence of HB vaccination uptake and associated factors among clients attending Budwale Health Centre in Mbale District. The results of the study will enable the facility to lay strategies to address the barriers of HB vaccine uptake and will also enable the policy makers to strategies to achieving the WHO targets of eliminating HBV infection. It will also add information to the existing knowledge on HB vaccination uptake and the associated factors among the general population.

## METHODOLOGY

### Study Setting

The study site was Budwale Health center III in Mbale District which is located in the eastern region of Uganda. It is found in East Africa approximately 225kms (140mile) northeast of the Kampala Capital City. According to the Uganda Bureau of statics, its population in 2019 was estimated to be 568,000 people, with 270,400 males and 298,400 females. The main economic activity is agriculture, farming in coffee, beans, Matooke, onions among others. Mbale is also a commercial and administrative city situated along a highway that joins Kenya to the Democratic Republic of Congo and South Sudan through Uganda. Mbale District has people of multi ethnic, cultural, social, and economic backgrounds. They practice a famous cultural and traditional Male circumcision (Imbalu) that does not employ safe medical practices. This puts the community at risk of acquiring blood-borne diseases including HBV infection.^20^

The district has 12 Government Health centers level II, 17 Health Centers level III at the county level, and 4 health centers level IV at sub-district with 2 hospitals. There is a Regional Referral Hospital (Mbale) with 332 beds. More so, it has 4 private/NGO dispensaries, and 7 health centers (III). Mbale district is said to have 4% prevalence of HBV infections among the blood donors. The study was conducted at Budwale Health Center which is one of the public health facilities located in Mbale District. The facility receives on average about 30 clients a day equivalent to about 10,950 annually for both inpatient and outpatient clients. All the services at the facility including immunization are free of charge.

### Study Design

Across sectional study design using quantitative methods of data collection was conducted between July and September 2022. Data on participants' demographic characteristics, HB vaccination status and other variables was collected using a questionnaire.

### Study Population

The study population included adult males and females who were above 18 years of age and in good health (not emergency cases) attending outpatient departments, ANC, Immunization and all other serving points at, Budwale Health Center III.

### Inclusion and Exclusion Criteria

Adult clients attending OPD and other service departments who were willing to participate in the study and meet the selection and sampling criteria were included in the study after consenting.

### The Exclusion Criteria:

Clients who needed immediate, urgent, and emergency care services were excluded from the study because the questionnaire needed some time to be completed. Even those who declined or who were not willing to participate were excluded from the study.

### Sample Size Determination

The sample formula by Daniel, 1999 was used to determine the sample size.^21,22^ With hepatitis vaccination uptake of 58%^23^, considering 95% confidence interval and margin of error of 5%, the calculated sample size was 375. taking into account 10% non-response, the sample size was increased to 412.

### Sampling procedure

All the 5 service provision sites of the facility were considered. These include inpatient wards, OPD, Antenatal Clinic (ANC) and Immunization Department. The number of participants to be picked from each site were determined proportionate to the sits client load while avoiding double counting of those who accessed more than one service. Simple random sampling was used to recruit the study participants at each unit. Printed papers with documentation of YES or NO in equal numbers were placed in opaque envelopes. These were reshuffled each morning and placed at the reception for each unit. All eligible participants were requested to pick one envelope and those who picked yes were taken through the consent process for recruitment while those who selected NO were dropped. This was done until the particular sample size for each unit was reached but avoiding duplication in case of multiple visits.

### Study Variables Dependent Variable:

Vaccinated (Vaccination status) all those who had received three doses were regarded as fully vaccinated. Those who had received 1 or two doses were regarded as partially vaccinated and those who had not received a single dose were regarded as not vaccinated. Vaccination status was measured by self-reporting of the study participants. Vaccination status / prevalence / up take was measured as the number of HB vaccine doses received.

### Independent Variables: Demographics

Age (was recorded as the age in complete years), sex (was recorded as participant's gender as either male or female), Education status (was recorded as none, primary, secondary or tertiary levels of education), source of income (was recorded as participant's occupation as formal employment or informal employment including farming business among others), religion, marital status, knowledge level on HBV and HB vaccines

### Data Collection Procedure and Tool

A structured questionnaire was pre-tested by administering it to 10 OPD clients at Budwale Health Center and issues raised were considered and corrected.

Research Assistants that included health workers (nurses, midwives, clinicians, and medical record officer) at Budwale Health center were inducted and trained on administering the questionnaire. The induction included reading through and interpreting the questionnaire into the local language.

### Data Management and Analysis

Data cleaning and validation was done and data was entered directly into SPSS and analysis was done using SPSS version 20. All socio-demographic variables were described by percentage, mean, and standard deviation. Bivariate analysis was done using cross tabulation and chi-square was used as a measure of association. Results of data analysis were presented in tabulated forms.

### Data Analysis Plan Prevalence of Hepatitis B Vaccination Uptake

Using self-reported vaccination status and the number of doses received. The frequency of those who had received one dose, two doses three doses and zero doses of HB Vaccine was determined and presented in numbers and percentages. Data was then coded in such a way that those who had received three doses of the vaccine were coded 1(vaccine uptake) and all others were coded 0. This enabled us to determine the proportion of vaccine uptake.

### Assessing Knowledge of Clients on HBV Infection and Vaccination

Participants were expected to respond to all the answers. Questions on the causes of HBV infection had a maximum of 5 correct responses and maximum score of 5. Questions on prevention had a maximum score of 5 for all the 5 correct responses. Questions on Signs and symptoms had a maximum score of 4 for all the correct responses. Questions on the effectiveness of the vaccine had maximum score of 2 for all the correct response. The recommended doses of the vaccine as well as the duration of coverage each had maximum score of 1. All correct responses were coded 1 and the wrong responses coded 0. Summation of total score for each respondent was then made. The total score for all the knowledge questions both HBV infection and vaccine was 18 (100%). Using blooms' score cut offs (Seid et.al 2018) we categorized knowledge as good knowledge for a score of 14 and above (80% and above), moderate knowledge, a score of 10–13 (60–79%) and poor knowledge a score of 9 (59%) and below. Each of the knowledge categories were then tested for association with vaccination uptake.

### Factors associated with vaccination uptake and the reason for not vaccinating

Descriptive statistics were used to present the frequencies of the factors associated with HB vaccination in terms of percentages. The results were presented in a table form. Bivariant analysis was done using chi-square to determine the factors that were independently associated with the outcome. This was independently applied on all the demographic characteristics, other factors and HB vaccination uptake. The variables that had twoby-two categories and meet the criteria, Pearson's chi-square values were used. Those two-by-two categorical variables that did not meet the criteria (assumption). For multi categorical variables the likely hood value was used to determine the level of significance. In all cases the level of significance alpha was taken to be 0.05. All factors whose level of significance was less than (< 0.005) were taken to be statistically associated with HB vaccination. There was no association between the factors and the HB vaccination uptake for all those factors whose level of significance was greater than 0.05. The measures of association between demographic characteristics, the levels of knowledge and the vaccination uptake were made using modified poison regression model.

### Ethical Consideration

The study was approved by the Makerere University School of Public Health Research and Ethics committee under the research protocol number MakSPH-REC O61. Permission from the in charge Budwale Health center was also sought before data collection. All participants were made to sign a consent form before participating. Confidentiality and privacy were also ensured.

## RESULTS

### Demographics

The study targeted 412 participants of which (97.8%)403 accepted to participate in the study and (2.2%) participants declined. Of those that participated, 50.4% (202) were male and 49.6% (199) were females. The mean age of respondents was 33years mean (SD)9.9. Majority of the respondents 71.0% (284) were married, 42.0% (166) had an education level of tertiary. Majority of the respondents were Muslims at 62.3% (251) followed by Catholics at 19.9% (80). A moderate number of the respondents 34.5% (139) were doing business and 32.5% (131) were in formal employments (Table 1).

**TABLE 1:** Demographic Profile of the Study Participants

Variable	Frequency	Percentage
Gender n= 401		
Male	202	50.4
Female	199	49.6
Marital status n=398		
Married	284	71.4
Single	93	23.4
Others	21	5.2
Education n=395		
None	24	6.1
Primary	91	23.0
Secondary	114	28.9
Tertiary or higher	166	42.0
Religion n=399		
Muslim	251	62.3
Catholic	80	19.9
Anglican	34	8.4
Born again	31	7.7
Others	3	0.7
Occupation and source of income n=399		
Farming	98	24.3
Business	139	34.5
Employed (formal)	131	32.5
Any other	31	7.7
Age Category n=402		
18–27	121	30.1
28–37	164	40.8
38–47	84	20.9
48–57	16	4.0
58 and above	17	4.2

HB vaccination uptake was at Sixty-eight-point seven percent 68.7% (277) of the respondents who had received three doses of HB vaccine. Eighty-six-point four percent 86.4% (344) of the participants had received at least one dose of the HB vaccine and 13.1% (52) had not received a single dose of the vaccine. Majority of the respondents 77.2% (274) had received their last dose of the vaccine more than 6 months back from the time of data collection (Table 2).

**TABLE 2:** Hepatitis B Vaccination Uptake

	Frequency	Percentage
Received three doses of HB vaccine	277	68.7
Received two and less doses of HB vaccine	126	31.3
Received 1 dose	18	5.1
Received 2 doses	54	15.3
3 doses	277	78.5
Received more than 3 doses	4	1.1
Vaccinated less than 1 months	13	3.7
Vaccinated within 1–3 months	32	9.0
Vaccinated 4 to 6mnths ago	36	10.1
Vaccinated more than 6 months ago	274	77.2

### Knowledge about Hepatitis B Virus Infection and Hepatitis B Vaccine

We asked respondents about the causes, prevention, the signs and symptoms of HBV infection. We also asked about the effectiveness of the HB vaccine, the number of doses and the duration of coverage of the vaccine. A moderate number of the respondents 183 (45.4%) responded that HBV can spread through body fluids contaminated with blood of an infected people. The majority 378 (95.8%) were aware HBV spread can be prevented by vaccination and 256 (66.5%) were able to tell that stomach pains and swelling and yellow eyes are signs and symptoms of HBV infection. Eighty-seven percent (87.1%) were aware that the vaccine is effective and the majority 268 (70.0%) were aware of the total number of doses for HB vaccinations. Using blooms score cut offs, knowledge score was further categorized into good knowledge (80% and above), moderate knowledge (60–79%) and poor knowledge (59% and below). The results showed that 144 (35.73%) had good knowledge, 209 (51.86%) had moderate knowledge and 50 (12.41%) had poor knowledge on both HBV infection and vaccination (Table 3).

**TABLE 3:** Knowledge on Hepatitis B Infection and Vaccination

Variable	Responses	Frequency	Percentage
How can Someone be infected with hepatitis B virus	through contact with the saliva of an infected person	167	41.4
	body fluids contaminated with blood of an infected person	183	45.4
	contact with sweat	171	42.4
	I don't know	51	12.7
How can we prevent Hepatitis B virus infection	through vaccination	378	95.8
	avoiding sharing sharp objects	216	54.3
	avoiding multiple sexual partners	98	24.6
	Sleeping under mosquito nets	2	0.5
	Use of facemasks	15	3.8
What are the signs and symptoms of HBV infection?	Headache, fevers, joint pains, and general body weakness	146	37.6
	Stomach swelling, stomach pains and yellow eyes	256	66.5
	Cough skin rash and excessive sleeping	18	4.1
	I don't know	37	9.6
How effectively do you think HB vaccine can protect people against HB infection?	Not effective	19	4.9
	Slightly effective	169	43.4
	Very effective	170	43.7
	I don't know	31	8.0
What do you think is recommended full dose of the hepatitis B vaccine?	1 dose	6	1.6
	2 doses	56	14.6
	3 doses	268	70
How long does a full dose of Hepatitis B Vaccine protect someone?	Less than a year	39	11.3
	1 year to 5 years	33	9.5
	6 years to 10 years	15	4.3
	11 years to 19 years	4	26.3
	20 years or more	114	32.9
	I don't know	141	40.8

### Bivariable and Multivariable Analysis for the Association between Bivariate Demographic characteristic, Knowledge and vaccination Uptake

In bivariable and multi variable analysis we used modified poison regression model to determine the association between vaccination uptake and the demographic characters as well as knowledge levels. The results revealed that clients aged 28 to 37 years were 21% more likely to be vaccinated against HB than those aged 18 to 27 years, PR=1.21, 95% CI: (1.025, 1.418), P=0.024. Besides, clients who had moderate knowledge were 99% more likely to be vaccinated against HBV compared to clients with poor knowledge, PR=1.99, 95% CI: (1.276, 3.106), P= 0.002. Additionally, clients with good knowledge about HB vaccination and infection were 2.8 times more likely to be vaccinated compared to clients with poor knowledge, PR=2.81, 95% CI: (1.830, 4.306), P<0.001. However, clients with primary and tertiary levels of education were less likely to be vaccinated against HBV than clients with non-formal education, PR=0.72, 95% CI: (0.598, 0.858), P<0.001 and PR=0.67, 95% CI: (0.574, 0.789), P<0.001, respectively. Furthermore, clients engaged in informal employment were 16% less likely to be vaccinated against HB compared to clients with formal employment, PR=0.84, 95% CI: (0.736, 0.953), P=0.007. Table 4

**TABLE 4:** Distribution of Participants according to the level of Hepatitis B Vaccination Knowledge

Knowledge category	Frequency	Percentage
Poor knowledge	50	12.4
Moderate knowledge	209	51.9
Good knowledge	144	35.7

In multivariable analysis, clients aged 28–37 years were 19% more likely to be vaccinated against HB compared to those aged 18–27 years, aPR=1.19, 95% CI: (1.026, 1.387), p=0.022. Clients with moderate knowledge about HB vaccination and infection were 96% more likely to be vaccinated against HB compared those with poor knowledge, aPR=1.96, 95% CI: (1.258, 3.051), P=0.003. Furthermore, clients with good knowledge about HB vaccination and infection 2.6 times more likely to be vaccinated against HB compared to those with poor knowledge, aPR=2.61, 95% CI: (1.701, 4.004), p<0.001. Clients with primary and tertiary levels of education were less likely to be vaccinated against HBV than clients with non-formal education, aPR=0.79, 95% CI: (0.662, 0.954), P=0.014 and aPR=0.69, 95% CI: (0.586, 0.808), P<0.001, respectively. Lastly, clients engaged in informal employment were 16% less likely to be vaccinated against HB compared to clients with formal employment, aPR=0.86; 95% CI: (0.764, 0.975), p=0.018. (Table 5).

**TABLE 5:** Bivariable and Multivariable Analysis for the association between Bivariate Demographic Characteristic, Knowledge and Vaccination Uptake

Variables	Un Adjusted PR	P val-ue	[95% C. Interval	Adjusted PR	P-value	[95% C. Interval
Sex						
Male	1.00			1.00		
Female	1.10	0.162	0.963, 1.254			
Marital status						
Single	1.00					
Married	1.30	0.07	0.76, 1.579			
Age groups						
18–27	1.00					
28–37	1.21	0.024	1.025, 1.418	1.19	0.022	1.026, 1.387
38–47	1.15	0.162	0.947, 1.386	1.06	0.531	0.881, 1.278
48–57	0.80	0.462	0.449, 1.439	0.90	0.699	0.512, 1.566
58+	0.87	0.588	0.514, 1.459	0.96	0.862	0.640, 1.452
Education Level						
None	1.00			1.00		
Primary	0.72	<0.001	0.598, 0.858	0.79	0.014	0.662, 0.954
Secondary	0.80	0.004	0.691, 0.934	0.87	0.094	0.744, 1.023
Tertiary	0.67	<0.001	0.574, 0.789	0.69	<0.001	0.586, 0.808
Religion						
Christians	1.00			1.00		
Muslims	0.72	<0.001	0.642, 0.805	0.80	<0.001	0.705, 0.898
Occupations						
Employed	1.00			1.00		
Informal						
Employment	0.84	0.007	0.736, 0.953	0.86	0.018	0.764, 0.975
Knowledge						
Poor knowledge	1.00			1.00		
Moderate knowledge	1.99	0.002	1.276, 3.106	1.96	0.003	1.258, 3.051
Good knowledge	2.81	<0.001	1.830, 4.306	2.61	<0.001	1.701, 4.004

### The Participants Reasons for Non-Vaccination

We examined participants' reasons for not vaccinating and the following were the outcomes: 11/52(21.2%) were not aware of HB vaccination, 9/52(17.3%) reported that they did not have time to go the vaccination. 15/52(28.8%) were afraid of contracting the virus from the vaccine and 2% thought that the vaccine was expensive. Concerning the health facility factors, we asked participants about the availability of logistics when they visited the health facility, 46(11.5%) agreed that the logistics were not available at the facility, 335(83.5%) disagreed and the rest were undecided. There were 336(95.1%) who agreed that whenever they went to the health center there were health workers. The rest disagreed or were undecided. About the long waiting ques at the health facility that would stop clients from getting vaccinated, only 27(6.8%) agreed all the rest disagreed or were undecided. Participants were asked whether their occupations put them at risk of contracting hepatitis B virus infection 159(39.9%) reported that they had no risk, 69(17.3%) reported low risk, 67(16.8%) reported moderated risk, and 59(14.8%) had a high risk of exposure. 14(11.1%) did not know. To determine the factors that are independently associated with hepatitis B vaccination, a chi square analysis was conducted at a level of 95% confidence. Vaccines being expensive (P=0.004), availability of vaccines and other logistics at the health facility (P=0.000), presence of health workers at the health facility (P=0.000), the distance of community members to the health facility (p=0.001), long waiting ques at the facilities (p=0.001) and occupational risks (0.000) were independently associated with HB vaccination status. Binary logistic regression revealed that all the above factors were negatively associated with vaccination uptake. The summary of the factor associated with HB vaccination is as illustrated in Table 6.

**TABLE 6:** Participants Reasons for Non-Vaccination

Variables	Responses	Frequencies	Percentage	Chi Square value	P value
Why have you not received a hepatitis B vaccination N=52	I am not aware of HB vaccination	11	21.2	5.768	0.800
	I don't know where to go for vaccination	5	9.6	0.000	1.000
	I don't have time		2.2	1.234	0.267
	Am afraid of contracting the virus from the vaccine	15	3.7	0.025	0.875
	The vaccine is expensive	1	1.9	11.858	0.004
	I don't see the need	11	21.2	0.694	0.405
	**Health Facility Factors**				
Vaccines and other utilities not available at health facility	Strongly agree	30	7.5	97.297	0.000
	Agree	16	4.0		
	Undecided	6	1.5		
	Disagree	173	43.1		
	Strongly disagree	162	40.4		
	Not applicable	14	3.5		
No health workers at the vaccination unit	Strongly agree	8	2.0	42.924	0.000
	Agree	4	1.2		
	Undecided	5	1.2		
	Disagree	134	33.3		
	Strongly disagree	249	61.8		
	Not applicable	7	1.7		
Health facility is too far	Strongly agree	6	1.5	29.650	0.001
	Agree	5	1.2		
	Undecided	7	1.7		
	Disagree	229	56.8		
	Strongly disagree	146	36.2		
	Not applicable	10	2.5		
Long que for vaccination	Strongly agree	8	2.0	30.014	0.001
	Agree	19	4.8		
	Undecided	4	1.0		
	Disagree	216	54.7		
	Strongly disagree	134	33.9		
	Not applicable	14	3.5		
perceived risk of occupational exposure to HBV infection	No risk of exposure	159	39.9	37.688	0.000
	Low risk of exposure	69	17.3		
	Moderate risk of exposure	67	16.8		
	High risk of exposure	59	14.8		
	I don't know	44	11.1		

## DISCUSSION

Hepatitis B vaccination uptake was at 68.7% which was lower than the desired percentage of about 95% of the community to achieve herd immunity. This therefore meant that more efforts are still needed to achieve immunity of the community against HBV infection. These findings were similar to the findings among commercial sex workers in Netherland and among the health care workers in wakiso.^15,16^ This uptake was however higher than those among health workers in Juba, among adult patients in south Poland, among the medical students at Makerere and those at the national hospital in Tanzania.^8,9,11,14^

This low to moderate uptake hepatitis B vaccination can be attributed to the sensitization campaigns by the government and the availability of free vaccines. The study findings on knowledge, were slightly different from those among the health workers in lagos.^16^ This may be due to the fact that both the previous studies were conducted among health workers. Thus, by nature of their occupation and profession are expected to at least have a minimum level of knowledge on the hepatitis B virus infection and vaccination. The findings therefore imply that more sensitization campaigns are needed among the community members on both Hepatitis B infection and vaccination..

According to the study findings, few participants reported being unaware of hepatitis B vaccination (21.2%) very few reported having no time to go for vaccination (2.2%) and very few participants were afraid of contracting the disease from the vaccines (3.7%). The findings were far different from the earlier studies of Enugu Nigeria that highlighted lack of time as one of the reasons for low vaccination prevalence.^13^ This partly explains why the current study found high prevalence of HB vaccination, implying that the community was informed of the vaccination and this partly explains why people have responded to the call of being vaccinated.

The study further found that a small percentage of participants (11.5%) agreed that when they visited the health facility there were no vaccines and logistics compared to (83.5%) that reported finding vaccines and logistics at the health facility. This explains the moderately high vaccination prevalence of above 78% in this study. However, the findings were different from the findings of Omotowo and others that found the cost of vaccines and long waiting queues as the reasons for the low prevalence of hepatitis B vaccination in Enugu Nigeria.^13^

Presence of health workers at the health facility (95.1%), being in close proximity with the health facility (93.0%) and not finding long waiting queues (88.6%) would also explain why the prevalence of the HB vaccination was moderately high in this study compared to a study by Atiba and others, Omotowo and others as well as Azado and others that reported long waiting queues, long distance from the health facility and absence of health workers at the health facility as major factors for low prevalence of hepatitis B vaccination.

## CONCLUSIONS AND RECOMMENDATIONS

The hepatitis B vaccination uptake coverage was lower than that required to achieve community herd immunity. Knowledge about Hepatitis B infection and vaccines, informal employment, age, and education were associated with vaccination uptake. This means that improving on the knowledge and awareness of the community about hepatitis B infection and vaccination would significantly improve on the uptake of hepatitis B vaccination. Cost of vaccines, presence and availability of health workers at health facilities, the distance from the health facility, long waiting queues and perceived occupational risks were reasons for low uptake of HBV vaccine.

The study recommends that availing free vaccines and other logistics, empowering health facilities and health workers can improve vaccine uptake by the community. Secondary, improvement on the education, sensitization and awareness campaigns by the DHTs and the health sub districts up to the lowest levels to improve on the community's knowledge on both hepatitis B infection and HB vaccination.

### Study limitations

Due to self-reporting there is a possibility of recall bias. Data was collected from clients attending Outpatient Department and other service units of the health facility. These have been selected to represent the general community that interacts with health workers (which is a high-risk group). Therefore, the results may not be easily generalized to the whole community. This was minimized by calculating and collecting data from an adequate and appropriate sample size. The participants are already sick (patients) and therefore at a risk of HB comorbidity and not a true representation of the community.
